# Histomorphometric study on blood cells in male adult ostrich

**Published:** 2013

**Authors:** Mina Tadjalli, Saeed Nazifi, Behrokh Marzban Abbasabadi, Banafsheh Majidi

**Affiliations:** 1 *Department of Basic Sciences, School of Veterinary Medicine, Shiraz University, Shiraz, Iran; *; 2 *Department of Clinical Studies, School of Veterinary Medicine, Shiraz University, Shiraz, Iran.*

**Keywords:** Blood cells, Histomorphometry, Iran, Ostrich

## Abstract

In order to perform a histomorphometric study of blood cells in male adult ostrich, blood samples were obtained from jugular vein of 10 clinically healthy male adult ostriches (2 - 3 years old). The slides were stained with the Giemsa methods and the smears were evaluated for cellular morphology, with cellular size being determined by micrometry. The findings of this study revealed that the shape of the cell, cytoplasm and nucleus of erythrocytes in male adult ostriches were similar to those in other birds such as quails, chickens, Iranian green-head ducks.

## Introduction

Although serology is the predominant method of disease monitoring in commercial poultry, examination of blood smears, bone marrow and clinical chemistry values are rarely done.^1^ Chicken has been used as the research animal model for establishing normal parameters for other species, but characteristic features of normal blood cells have been studied by light microscopy in other domestic birds, too.^2^^-^^12^ However, there is a little information available on the blood cell morphology of adult male ostriches. So the object of this study was to characterize the morphology of adult male ostriches.

## Materials and Methods

Blood samples were obtained from 10 clinically healthy male adult ostriches (2 - 3 years old). The samples were taken with syringes gauge 14, from the jugular vein and collected into tubes containing EDTA. At least ten air-dried blood smears were prepared from each sample. All birds were confirmed as being free of hematological abnormalities following routine evaluation of the peripheral blood. The slides were stained with the Giemsa and Wright methods, and the stained smears were evaluated for cellular morphology by light microscopy and with cellular size being determined by micrometry.

## Results

**Fig. 1:**
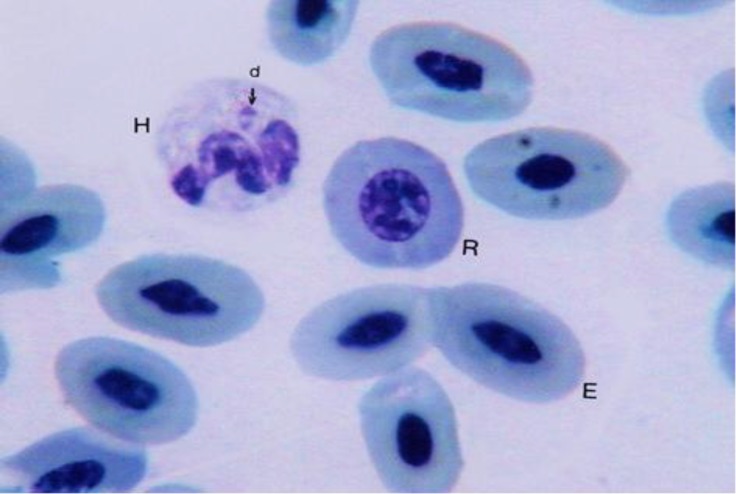
Erythrocyte (E); polychromatic erythrocyte (R); heterophil with drum-stick process that are joined to one of the nucleus segments (H and d), (Giemsa, 1800×).

**Fig. 2:**
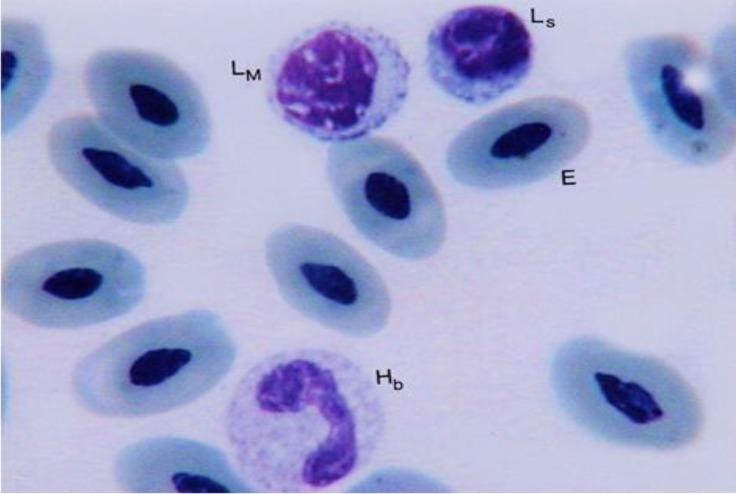
Lymphocytes in two different sizes; small (L_S_), and medium (L_M_); erythrocyte (E); heterophilic band (H_b_), (Giemsa, 1800×).

**Fig. 3:**
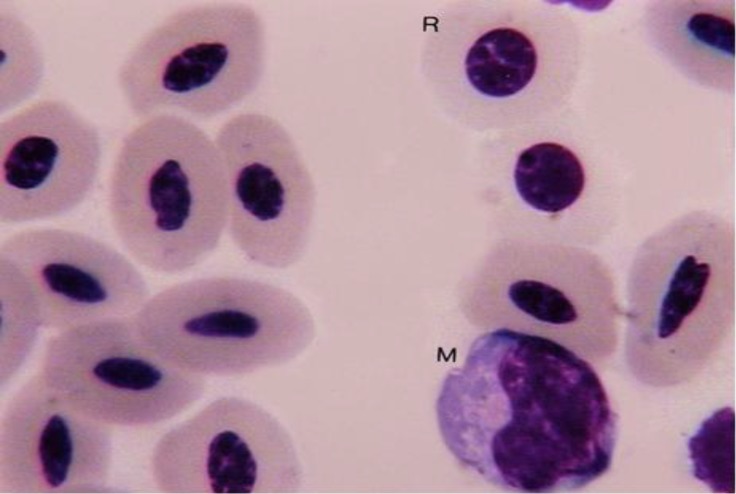
Monocyte (M); polychromatic erythrocyte (R), (Giemsa, 1800×).

**Fig. 4.**
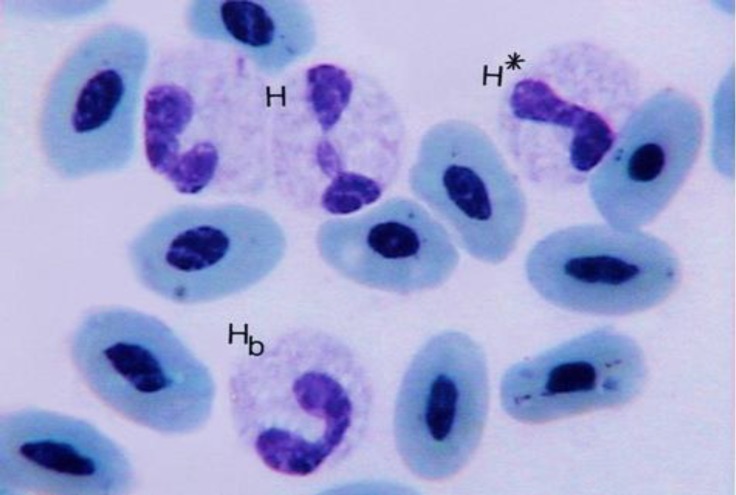
Heterophil (H); heterophilic band (H_b_); heterophil with non-segmented nucleous (H*) (Giemsa, 1800×).

**Fig. 5:**
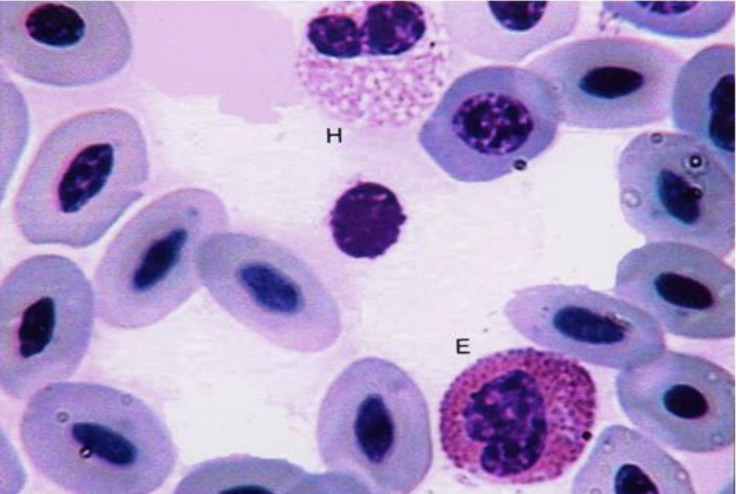
Eosinophil (E) and heterophil (H) (Giemsa, 1800×).

**Fig. 6:**
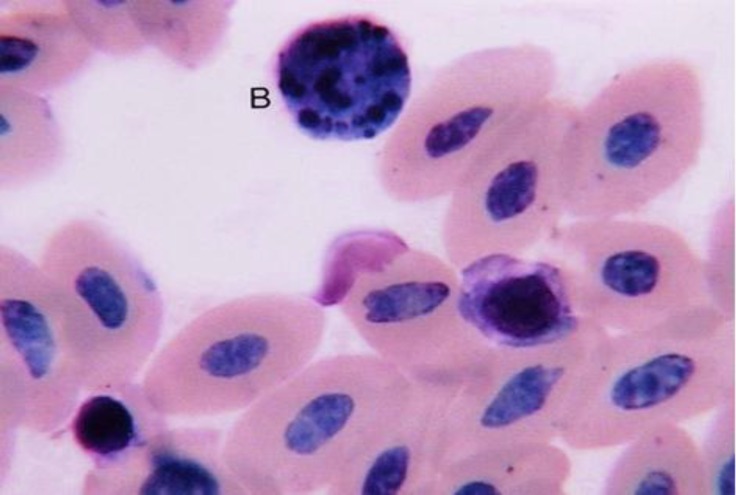
Basophil (B), (Giemsa, 1800×).

**Fig. 7:**
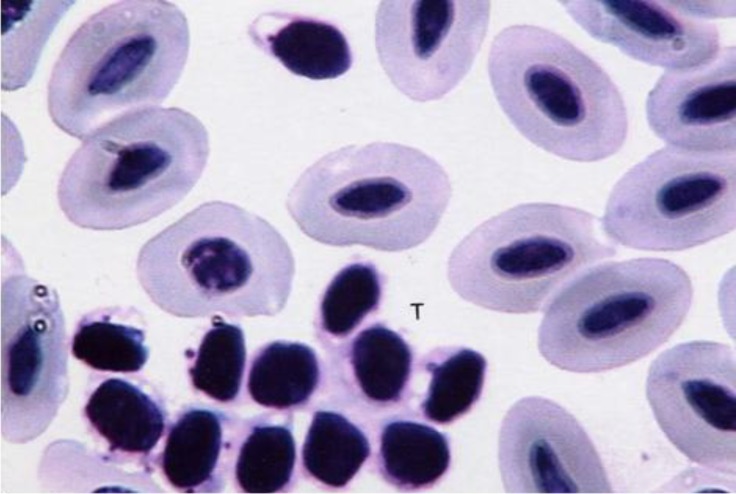
Thrombocytes (T) were seen individually or in groups, (Giemsa, 1800×).

The morphology of the cellular elements in the blood of male adult ostrich is presented in Figs. 1-7.

Erythrocytes were oval-shaped with two round extremities (Fig. 1). The basophilic nucleus was oval-shaped with two round and/or sharp extremities. It contained reticular chromatin but had no nucleolus. The nuclei were located in the central part of the cell. The cytoplasms of the erythrocytes were light blue to gray.

Reticulocytes in male adult ostrich were seen among the erythrocytes (Figs. 1 and 3). Morphologically, the reticulocytes were shorter than erythrocytes. The reticulocytes were oval-shaped but the nucleus was wider and lighter than the erythrocytes.

Lymphocytes were round and contained a large rounded nucleus with dense chromatin (Fig. 2). The nucleus may show a small depression and fills nearly the entire cell, leaving only a narrow rim of cytoplasm. The lymphocytes were seen in two different sizes and were classified as medium or small. The medium size lymphocytes had more cytoplasm than the small size. Lymphocytes were approximately the same size as heterophils.

Monocytes were large round cells which contained a large polychromatic oval-shaped nucleus (Fig. 3). The nucleus was located in one side of the cell. In some monocytes the nucleus was bean-shaped. The cytoplasm was basophilic and had a vacuolated appearance. Monocytes were the largest leukocytes in male adult ostrich.

Heterophils were round cells containing a lobulated nucleus, which had two or three segments, although nucleus with four segments was observed (Figs. 2, 4 and 5). The nucleus segments were round and/or oval-shaped with different sizes. Organization of these segments caused the heterophils nucleus to change to different forms. Occasion-ally, some heterophils showed the non-segmented nucleus connected, it might be like the band cell, but in band cells, nucleus was completely horseshoe-shaped. The cytoplasm was large and had pink to purple specific rounded granules. A very small nucleus process named drum-stick was seen in a few of the ostrich heterophils that was joined to one of the nucleus segments (Fig. 1). Heterophils were the main leukocytes in the blood cells of male adult ostriches.

Eosinophils were round cells with a two or three segmented nucleus (Fig. 5). The nucleus segments were rounded or oval-shaped in various sizes. The cytoplasm was large and occupied by small specific acidophilic granules. The granules were orange to red. 

Basophils were round cells with a segmented spherical nucleus located in one side of the cytoplasm (Fig. 6). The cytoplasm was low and contained specific highly basophilic granules. The granules were spherical and often covered the nucleus. The basophils in male adult ostrich were the fewer leukocytes.

Thrombocytes were seen individually or in groups. They were spherical or oval-formed with a central nucleus (Fig. 7). The shape of nucleus matched with cell shape. The nucleus contained heterochromatin with a little basophilic cytoplasm.

## Discussion

The findings of this study revealed that the shape of the cell, cytoplasm and nucleus of erythrocytes in male adult ostriches were similar to those in other birds such as quails, chickens and Iranian green-head ducks.^2^^,^^3^^,^^6^^,^^8^^,^^9^ The erythrocytes in male adult ostriches were 12.96 ± 0.92 µm in length and 8.62 ± 1.19 µm in width. It was 11.38 ± 0.76 and 6.97 ± 0.50 in black headed gull^15^ and 11.82 ± 0.03 µm and 6.47 ± 0.02 µm in Iranian green-headed ducks.^8^

The size of reticulocytes in male adult ostriches was similar to those in black headed gull and Japanese quail.^9^^,^^15^ The reticulocytes in male adult ostriches were 9.89 ± 3.26 µm in length and 7.78 ± 2.95 µm in width. The number of reticulocytes was 6.00 ± 0.70 percent of the entire peripheral blood cells. It is approximately similar to those in chickens and black headed gull.^2^^,^^15^

The morphology of eosinophils in male adult ostriches was similar to those in north Iranian green-head ducks, black headed gull, Japanese quail, domestic chicken and adult quail.^2^^,^^8^^,^^9^^,^^14^^,^^15^ The diameter of eosinophils was 11.57 ± 1.72 µm in male adult ostriches, in black headed gull the diameter was 10.67 ± 0.67 µm and in domestic chicken it was 7.30 µm.^2^^,^^15^

The number of eosinophils was 2.67 ± 0.57 percent of the entire white blood cells (WBC) in male adult ostriches and 2.58% of WBSs in emu.^16^ Tadajlli *et al.* reported that the number of eosinophils in north Iranian male green head ducks was 3.20 ± 0.44 percent of WBCs.^8^

The morphology of basophils in male adult ostriches was similar to those in black headed gull, chicken, Iranian green head ducks, adult quail, turkey, goose and guinea-fowl.^8^^,^^9^^,^^14^^,^^15^^,^^17^ The diameter of basophils in male adult ostriches was 10.89 ± 1.72 µm, 10.59 ± 0.69 µm in black headed gull,^15^ 7.17 ± 0.04 µm in Iranian male green head ducks.^8^ Basophils were the largest leukocytes in male adult ostriches, like basophils in adult quail,^9^ black headed gull^15^ and Iranian green head ducks.^8^ In male adult ostriches there were no basophils. It was 0.96 ± 0.73 percent of WBCs in black headed gull,^15^ and 1.90 ± 0.23 percent in Iranian green head ducks.^8^

The heterophils in male adult ostriches were similar to those in north Iranian green head ducks,^8^ black headed gull,^15^ domestic chicken,^7^^,^^18^^,^^19^ goose.^20^ Some heterophils showed a non-segmented nucleus, similar to those in Iranian green head ducks.^8^ The diameter of heterophils in male adult ostriches was 11.50 ± 1.02 µm, and in black headed gulls 10.68 ± 0.85 µm.^15^ The number of heterophils in male adult ostriches was 48.00 ± 14.91 percent of WBCs, and involved the highest number of WBCs in male adult ostriches. The number of these cells in black headed gull was 31.24 ± 8.47 percent,^15^ and 23.06 ± 7.45 percent in Iranian male green head ducks.^8^ Robertson and Maxwell reported that the number of heterophils were 74.80% of WBCs in adult ostriches.^21^ Stoskopf *et al.* reported that the number of heterophils was 77.91% of WBCs in adult ostriches.^22^

The morphology of lymphocytes in male adult ostriches was similar to those in black headed gull, domestic chicken, and north Iranian green head ducks.^2^^,^^8^^,^^14^^,^^15^ The diameter of small and medium lymphocytes in male adult ostriches was 7.33 ± 0.60 µm and 11.00 ± 1.70 µm, 6.57 ± 0.89 µm in black headed gull,^15^ 5.16 ± 0.04 µm in Iranian male green head ducks and 7.73 ± 1.33 µm in male adult quail^8^^,^^9^ The lymphocytes contained 46.80 ± 15.30 percent of leukocytes in peripheral blood cells. These cells were 27.00% of leukocytes in male adult ostriches and 59.00% to 80.00% in domestic chicken,^14^ and 61.00 ± 18.40 percent in great black backed gull.^3^ Robertson and Maxwell reported that the lymphocytes contain 14.00% of leukocytes in ostriches;^21^ also these cells contain 19.80% of leukocytes in emu and 19.70% in cassowary.^16^^,^^22^

The morphology of monocytes in male adult ostriches was similar to those in duck, turkey, goose, quail and pigeon,^17^ black head gull,^15^ chicken.^2^^,^^3^ In male adult ostriches the diameter of monocytes was 12.50 ± 1.30 µm, which is similar to those in black head gull.^15^ These cells were the largest leukocytes in male adult ostriches, similar to those in Iranian green head duck.^8^ These cells contained 3.60 ± 1.14 percent of leukocytes in male adult ostriches. These are 1.52 ± 1.12 percent in black headed gull and 9.33 ± 4.23 in male adult quail.^3^^,^^15^ Reportedly, monocytes contain 3.00% of leukocytes in adult ostriches,^21^ and these cells contain 0.10% of leukocytes in emu.^16^

Thrombocytes in male adult ostriches were spherical and/or oval in shape. Although these are spherical shaped with an occasional oval form in Iranian green head duck^8^ and black head gull,^15^ in chickens the shape was more often oval.^2^^,^^3^^,^^22^ The purple azurophilic granules were seen individually or in groups. The morphology of thrombocytes in male adult ostriches was similar to that in quail, goose, turkey, and pigeon.^17^ The diameter of spherical shaped thrombocytes in male adult ostriches was 6.18 ± 0.75 µm, although it is 4.06 ± 0.28 µm in black head gull and 4.41 ± 0.031 in Iranian male green headed duck.^8^^,^^15^ Tadjalli *et al.* reported that the diameter of thrombocytes in male adult quail was 4.10 ± 0.30 µm.^9^ The oval-shaped thrombocytes in male adult ostriches were 8.00 ± 1.60 µm in length and 4.56 ± 0.40 µm in width. The diameter of thrombocytes in chicken was 6.10 µm in length and 11.50 µm in width.^3^ The number of thrombocytes in male adult ostriches were 33.00 ± 8.80 (×10^3^ per µL) that is approximately similar to black head gull.^15^
